# Long term stability and infectivity of herpesviruses in water

**DOI:** 10.1038/srep46559

**Published:** 2017-04-21

**Authors:** Anisha Dayaram, Mathias Franz, Alexander Schattschneider, Armando M. Damiani, Sebastian Bischofberger, Nikolaus Osterrieder, Alex D. Greenwood

**Affiliations:** 1 Leibniz-Institute for Zoo and Wildlife Research, Alfred-Kowalke-Strasse 17, 10315 Berlin, Germany; 2 Institut für Virologie, Freie Universität Berlin, Robert-von-Ostertag-Str. 7-13, 14163 Berlin, Germany; 3 Department of Veterinary Medicine, Freie Universität Berlin, Oertzenweg 19b, 14163, Germany

## Abstract

For viruses to utilize environmental vectors (hard surfaces, soil, water) for transmission, physical and chemical stability is a prerequisite. There are many factors including pH, salinity, temperature, and turbidity that are known to contribute to the ability of viruses to persist in water. Equine herpesvirus type-1 (EHV-1) is a pathogenic alphaherpesvirus associated with domestic horses and wild equids. EHV-1 and recombinants of EHV-1 and EHV-9 are able to cause infections in non-equid animal species, particularly in captive settings. Many of the captive non-equid mammals are not naturally sympatric with equids and do not share enclosures, however, in many cases water sources may overlap. Similarly, in the wild, equids encounter many species at waterholes in times of seasonal drought. Therefore, we hypothesized that EHV-1 is stable in water and that water may act as a vector for EHV-1. In order to establish the conditions promoting or hindering EHV-1 longevity, infectivity and genomic stability in water; we exposed EHV-1 to varied water environments (pH, salinity, temperature, and turbidity) in controlled experiments over 21 days. The presence and infectivity of the virus was confirmed by both qPCR and cell culture experiments. Our results show that EHV-1 remains stable and infectious under many conditions in water for up to three weeks.

To date, nine equine herpesviruses have been described, including six that have been assigned to the subfamily *Alpaherpesvirinae* and three that are within the subfamily *Gammaherpesvirinae*. Equine herpesvirus type-1 (EHV-1) belongs to the *Varicellovirus* genus in the subfamily *Alphaherpesvirinae*. It is one of the most important pathogens of domestic horses worldwide^1^,^2^, with symptoms including spontaneous abortion, respiratory disease, myeloencephalopathy and neonatal foal disease^3^.

Although horses are natural hosts for equine herpesviruses, several viruses have recently been discovered that are closely related to EHV-1 and were isolated from both captive and wild animals, including Thompson’s gazelles (*Eudorcas thomsoni*)^4^, Ilamas (*Lama glama*)^5^, alpacas (*Vicugna pacos*)^6^, black bears (*Ursus americanus)*, polar bears (*Ursus maritimus*)^7^ and rhinos (*Rhinoceros unicornis*)^8^,^9^. Serological and genetic evidence suggests that zebra-conspecific and rhinos in Africa are frequently exposed to EHV-1 and its close relative EHV-9, which may even reservoir in rhinos (Abdelgawad *et al*. 2015). It is evident that cross species transmission of EHV-1-like viruses occurs in both captive and wild animals; however, the mechanism by which virus is transmitted is yet to be determined. As most captive animals do not have direct contact with equine species, it is most likely that viral transmission is indirect. Water bodies are a common feature of many zoos and in the environment and are often shared by many species. In the wild, many equid species and sympatric species live under conditions of seasonal water shortages forcing mass congregations of animals at residual water sources. Therefore, it is possible that water may act as an environmental vector mediating the transmission of EHV-1 in both captive and wild animal populations. A prerequisite to this hypothesis is that EHVs are stable in water. Environmental vectors in this study are defined as abiotic entities that indirectly facilitate the transmission of viruses.

Few studies have researched the stability of viruses in water under different environmental conditions; with most focusing on viruses that have a direct impact on humans (influenza, adenovirus, noroviruses, rotavirus and astrovirus)^10^,^11^,^12^,^13^ or animal health^14^,^15^. Many of the viruses examined are enteric viruses, which contaminate water rather than actively use water as a vector. Studies of viral stability have demonstrated that environmental factors such as temperature, humidity, salinity, pH, and ultraviolet light affect the time that enteric viruses remain infectious. Examining the impact of these variables on viral stability gives insights into whether water is likely to serve as an environmental vector for the virus to be transmitted to other potential hosts. We contend that water is a likely transmission vehicle used by many viruses to maintain themselves within populations.

We here investigate the stability of EHV-1 in spiked water samples. We establish the minimum detectable concentration of EHV-1 that is needed for infectivity and how long the virus remains infectious in water over a 3-week period. In addition, we examine different simulated environmental conditions, including pH, salinity, temperature and turbidity that are known to influence virion stability^16^,^17^. We employ cell culture and qPCR methods to determine how long EHV-1 remains infectious as a function of the different conditions applied.

## Material and Methods

A series of experiments were conducted to test the persistence and residual infectivity of EHV-1 at different concentrations. In addition, the tenacity of EHV-1 was examined under different environmental conditions: pH, salinity, temperature, and sediment concentrations over a 3-week period. The variables were selected as we were able to keep them constant for the 3-week period that the experiment would run and as we had observed their importance in environmental water sources utilized by equids (unpublished data not shown).

### EHV-1 preparation

The experiments were performed using the EHV-1 (Mar-87) strain originally supplied by P. Thein (München, Germany) and subsequently propagated on rabbit kidney (RK13) cells at stock concentrations of 10^5^ tissue culture infective doses (TCID_50_)/ml. A diluted viral suspension was prepared using Dulbecco’s Modified Eagle’s Medium (DMEM) (Life Technologies, California, United States) with 0.5% kanamycin and 2% fetal bovine serum (FBS) (Gibco, Europe). Two hundred microliters of EHV-1 solution were added to RK-13 cells grown in 10 ml of DMEM (2% FBS + 0.5% kanamycin). After complete cytopathic effect (CPE) was observed, EHV-1 was recovered by three freeze-thaw cycles, followed by centrifugation (3,000 × g for 15 min) to remove cellular debris^18^. The EHV-1 concentration was then determined by titration.

### EHV-1 spiking and serial dilution of distilled water

Three milliliters of titrated EHV-1 (10^3.5^ TCID_50_/ml) were added to 47 ml of double-distilled water (ddH_2_O). A 10-fold serial dilution from the initial 50 ml was generated. The 10-fold serial dilution was then left at room temperature (20 °C), and sampled on day 1 and every third day for the first week (Day 1, 3, 6 and 7) and every week thereafter for three weeks (Day 14 and 21). At each sampling point, 120 μl of sample was frozen at −80 °C for subsequent cell culture. DNA was extracted from 1 ml of each water sample.

### EHV-1 spiking: pH, salinity, temperature, and sediment concentrations in ddH_2_O

The stability of EHV-1 was tested under four different conditions: salinity, pH, temperature and sediment turbidity. A pH range (4, 6, [1 M HCl buffer] 8 and 10 [1 M NaCl buffer]) was adjusted in four 1 L bottles of ddH_2_O. Four different salinity concentrations in ddH_2_O were tested to mimic different environmental conditions: freshwater (50 parts per million [ppm] 0.05 g of NaCl), brackish (1,000 ppm 1 g of NaCl), salinic (3,000 ppm 3 g of NaCl) and sea water (35,000 ppm 35 g of NaCl). Three different temperatures were tested: 4 °C, 20 °C and 30 °C. Four different organic sediment (turbidity) concentrations were also tested. The sediment contained 33% organic substance, pH 5.5 (NaCl 1.5 g/L, N 250 mg/L, P_2_O_5_ 230 mg/L, K_2_O 320 mg/L, Mg 160 mg/L and S 120 mg/L). Four different dilutions of organic sediment were used: 7.5 g/L, 25 g/L, 50 g/L and 100 g/L. For each pH, salinity and sediment trial, the water (1 L) was inoculated with 63.8 ml of infectious EHV-1 (10^3.5^ TCID_50_/ml). For all treatments, only the water was sampled. In the water and sediment samples, no centrifugation step was necessary as the sediment had completely settled by the time of sampling. In this case, only water was sampled.

A second experiment with sediment and water was conducted. 1 L of ddH_2_O with four different weights of organic sediment was used: 7.5 g/L, 25 g/L, 50 g/L, and 100 g/L. Each treatment was spiked with 63.8 ml infective EHV-1 (10^3.5^ TCID_50_/ml). The surface water and sediment were sampled separately. Both water and sediment samples were taken at 1 day post inoculation (DPI) and every third day for the first week (DPI 1, 3, 6 and 7) and every week there after for up to three weeks (Day 14 and 21).

### EHV-1 cell culture

Monolayers of RK-13 cells at an approximate concentration of 10^7^ cells in 75 cm^2^ tissue culture flasks were treated with 1% trypsin-EDTA and 2X DMEM solution. 400 μl of the suspension (2 × 10^5^ cells/ml) was added to each well of 24-well plates. Cells were inoculated with 50 μl of the samples from the serial dilutions and pH, salinity and sediment concentrations. Duplicate plates were made for each treatment. After one hour, 200 μl of 1.5% carboxyl methyl cellulose (CMC) was added and cultures further incubated at 37 °C under 5% CO_2_ for 7 days. The first sets of plates were then stained with 10% formalin and 1% crystal violet to determine CPE and demonstrate the virus had remained infectious. From the second set of plates, the cells were removed with a scraper and viral DNA extracted for EHV-1 qPCR analysis.

### EHV-1 DNA isolation

Extraction of viral DNA from 1 ml of the ddH_2_O serial dilutions as wells as pH, salinity and sediment concentration samples were carried out using the Invisorb^®^ Spin Virus DNA Mini Kit (Stratec biomedical, Germany). The following modifications were made to the protocol: 400 μl of lysis buffer, 400 μl of binding buffer and 20 μl of proteinase K and carrier RNA were used per sample. Samples were eluted in 60 μl of elution buffer.

### Quantitiative PCR (qPCR) for detection of EHV-1

To quantitatively determine the presence of EHV-1 DNA from both the serial dilution and environmental conditions, qPCR was carried out. qPCR was performed on the DNA extracted after cell culture to ensure the CPE observed was indeed caused by EHV-1. The qPCR targeted and amplified a 106 base pair sequence of the glycoprotein B (gB) gene of EHV-1 (GenBank accession no. M36298) as previously described by Hussey *et al*. 2006^19^. The reaction contained 100 nM of the fluorogenic Taqman probe **6FAM**-TGA GAC CGA AGA TCT CCT CCA CCG A-**BHQ1**, 450 nM of the forward primer **5′**-CAT ACG TCC CTG TCC GAC AGA T-**3′** and reverse primer **5′**-GGT ACT CGG CCT TTG ACG AA-**3′**, 10 μl of the SensiFAST^TM^Probe Lo-ROX (Bioline, Germany) and 5 μl of the template DNA. The qPCR was carried out in 96 well microtitre plates using the Real Time PCR 7500 FAST System (Applied Biosystems, California, United States) under the following cycling conditions: 95 °C for 2 min, followed by 40 cycles of 95 °C for 3–10 s, 60 °C for 30 s and a final hold at 60 °C for 1 min. Each sample was run in triplicate and quantitated by regression to the slope of a standard curve generated from serial dilutions of isolated DNA from EHV-1 BAC, Ab4 strain^20^ (Supplementary Figure 1).

### Statistical analyses

The mean of triplicate Ct values for each sample was calculated and then the copy number was calculated using the equation Y molecules = (X g/μl DNA/[plasmid length bp × 660]) × 6.022 × 10^23^ where the EHV-1 pAB4 BAC is 157736 bp. The detection limit for EHV-1 with this assay was ~700 genome copies per μl.To evaluate virus tenacity, we fitted a linear model for each experiment with initial genome copy number (GCN) values as the response variable. Virus infectivity was assessed in two different ways: (1) by using linear models with GCN values from cell cultures as response, and (2) by using generalized linear models with binomial errors and with CPE results as response (coded as present and absent). In each model, we included day and treatment (i.e. dilution factor, pH level, salinity, and turbidity, respectively) as predictor variables. In addition, we initially included in all models the interaction between day and treatment, which accounts for the possibility that virus decay differs among treatment conditions. If an interaction effect was not statistically significant, it was removed from the model. Results for all linear models were inspected visually for assumptions regarding the normality and homogeneity of error variances. To prevent violations of these assumptions, we log-transformed GCN in all models. All analyses were performed in the statistical software R, version 3.2.4^21^.

## Results

### Stability of EHV-1 in distilled water

**Figure 1:**
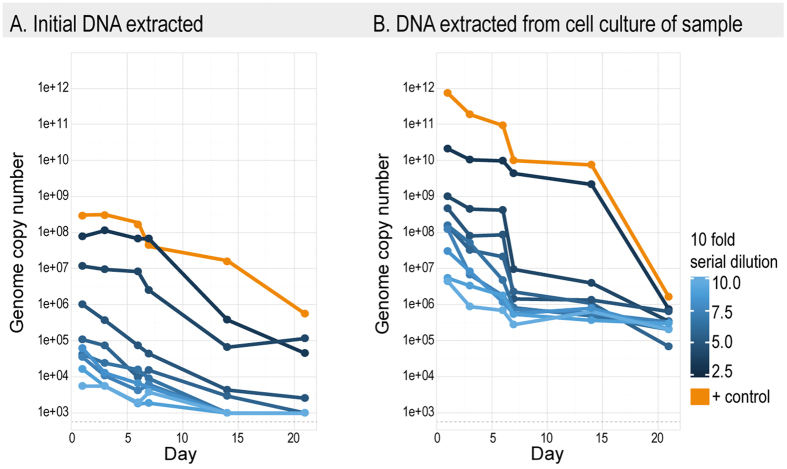
qPCR results for the detection of EHV-1 from DNA extracted from (**A**) the initial water (**B**) and post cell culture samples taken from 10-fold serial dilution of distilled water (1 L) spiked with EHV-1 (103.5 TCID50/ml). All values shown represent genome copy number (GCN) of EHV-1. Yellow lines indicate the positive control samples of EHV-1 in distilled water. Detection limit of EHV-1 is represented with a dotted grey line (~700). The experiment was run over 21 days at room temperature and samples were taken at six time points (days).

**Figure 2:**
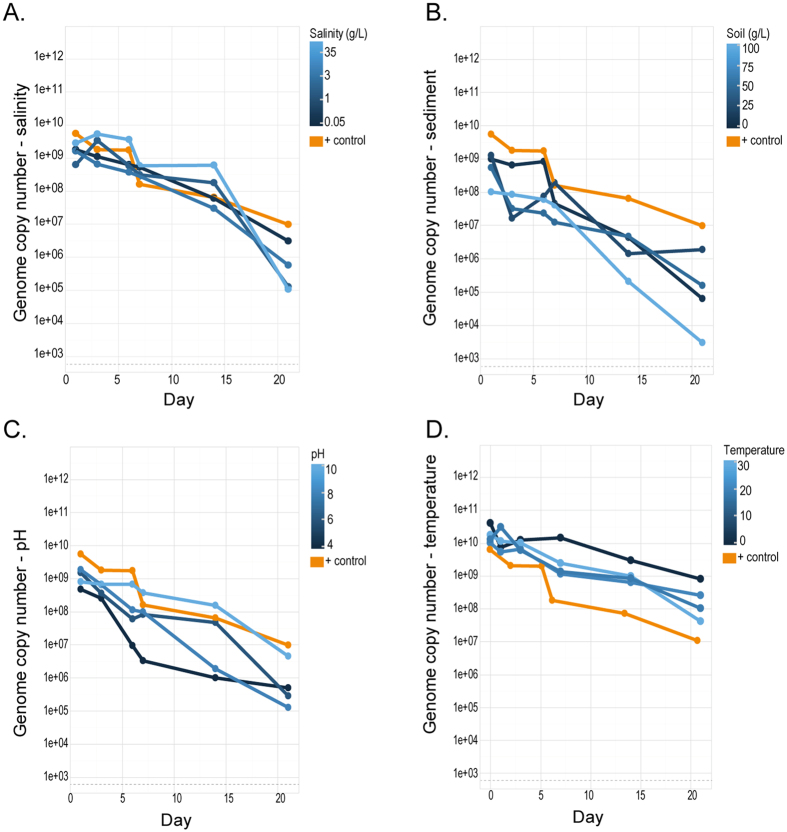
qPCR results for the detection of EHV DNA extracted from initial water samples taken from distilled water (1 L) under different conditions (**A**) salinity (0.05, 1, 3, 35 g/L), (**B**) sediment (7.5, 25, 50 and 100 g/L), (**C**) pH (pH: 4, 6, 8, 10), (**D**) temperature (4, 20, 30 °C) spiked with EHV-1(103.5 TCID50/ml). The experiment was run over 21 days and samples were taken at six time points (days). Yellow lines indicate the positive control of EHV-1 and distilled water. Detection limit of EHV-1 is represented with a dotted grey line. All values shown represent GCN of EHV-1.

EHV-1 was stable in distilled water for up to 14 days, as CPE were still detectable at this time point (Table 1). The overall trend for all dilutions of EHV-1 was a decrease in the DNA concentration (as measured by GCN) over the 3-week observation period (Fig. 1). In the samples with higher initial viral titers, EHV-1 DNA concentrations remained high for the first six days (Fig. 1). Statistical analysis supported both day and dilution factor as significant factors influencing virus concentration (p < 0.001 for both cases, Supplementary Table 1). The DNA concentration profiles were similar among dilutions, the more diluted samples exhibiting similar viral DNA concentrations for both the DNA extracted directly from water and extracted from cell cultures that were used to examine the EHV-1-spiked water dilutions over 21 days. This trend was also evident when determining dilutions that resulted in CPE, with CPE observed in the lower dilutions (10^−1^, 10^−2^) for the first 14 days (Table 1). The cell culture results also suggest that, regardless of the initial concentration of spiked EHV-1, no CPE was observed after 21 days (Table 1). Although we found a significant interaction between day and the dilution factor the rate of decay remained similar amongst samples of different dilution factors (Fig. 2). This suggests that the initial EHV-1 input concentration only persists up to 14 days in distilled water. The interaction between day and the dilution factor of the virus was significant in both the initial concentration and the qPCR post cell culture results (p = 0.038 and p < 0.001 respectively, Supplementary Table 1).

### Stability of EHV-1 under different pH conditions

**Figure 3:**
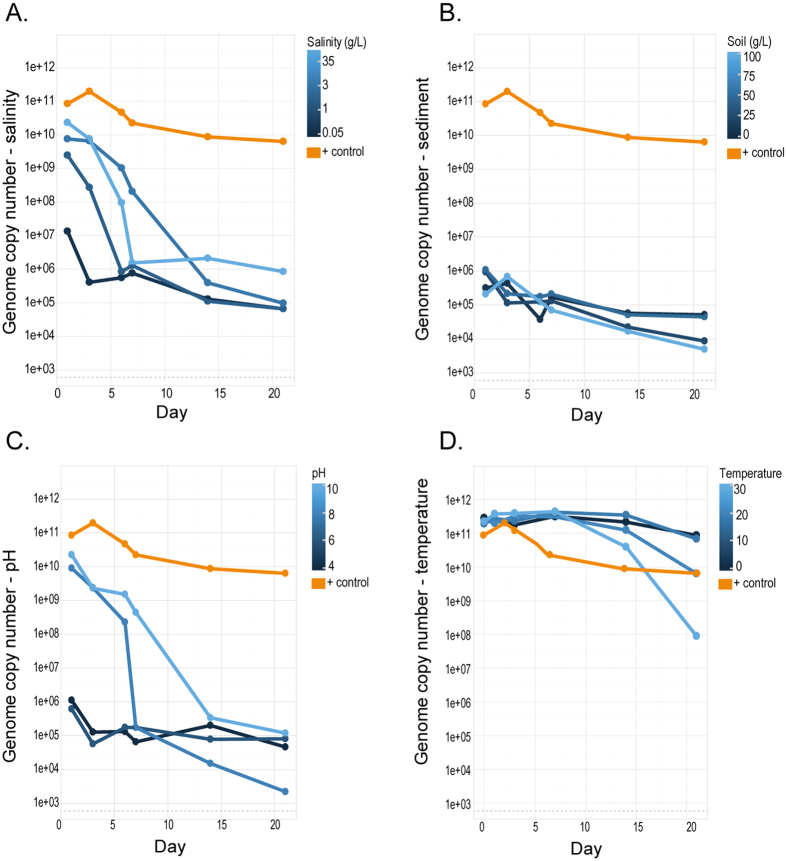
qPCR results for the detection of EHV- DNA extracted from post cell culture samples taken from distilled water (1 L) under different conditions (**A**) salinity (0.05, 1, 3, 35 g/L), (**B**) sediment (7.5, 25, 50 and 100 g/L), (**C**) pH (pH: 4, 6, 8, 10), (**D**) temperature (4, 20, 30 °C) spiked with EHV-1(103.5 TCID50/ml). The experiment was run over 21 days and samples were taken at six time points (days). Yellow lines indicate the positive control of EHV-1 and distilled water. Detection limit of EHV-1 is represented with a dotted grey line. All values shown represent GCN of EHV-1.

Changes in pH levels affected virus stability, but the specific effects notably differed between the water extractions and those from cell culture (Fig. 2A). EHV-1 DNA concentrations at higher pH (pH 8 and 10) was higher for DNA extracted from water but degraded rapidly after the first six days with both day and pH having a significant influence on stability (p < 0.001, Supplementary Table 1). The cell culture results (Table 2) (Fig. 3A,C) were consistent with the extracted water as the higher pH treatments (pH 8 and 10) resulted in higher EHV-1 DNA levels from the extracted cell cultures (GCN 2.3 × 10^8^−2.22 × 10^9^) for the first six days and induced CPE for up to 21 days, extending beyond what was observed in the dilution experiments at neutral pH. However, in contrast to the results of the DNA extracted from the initial water (Fig. 2C), EHV-1 concentrations at higher pH treatments decreased at a higher rate than lower pH treatments in cell culture (Fig. 3C), which resulted in similar concentrations (GCN 2,180–11,953)-) for all treatments by the end of the experiment (as indicated by a significant interaction between pH treatment and day, p = 0.001, Supplementary Table 1) (Fig. 3C). However, cell culture results (Table 2) show the remaining EHV-1 particles in the high pH treatments (pH 8 and 10) are still infectious up to 21 days.

### Stability of EHV-1 under different salinity conditions

EHV-1 concentrations in all five salinity conditions from extracted water initially varied but were relatively high (GCN 7.6 × 10^9^–8.63 × 10^10^). However, the highest salinity (35 g/L) had the highest initial concentration (day 1) but was the fastest to degrade (as indicated by a significant interaction between day and salinity treatment, p = 0.019, Supplementary Table 1) (Fig. 2A). CPE was observed in samples taken from preparations with the three highest salinities (1.0, 3.0 and 35.0 g/L) for up to 14 days DPI (Table 2). The qPCR post cell culture results illustrate both predictor variables day and salinity as being significant (p = 0.025 and p < 0.001) as both lower salinities (0.05 and 1.0 g/L) consistently had lower GCN recovered (Fig. 3A). This was similar to the observed CPE, where salinity had statistically significant effect on CPE (p = 0.03) and only found a significant effect of day (p = 0.048) (Supplementary Table 1).

### Stability of EHV-1 under different temperature conditions

A decrease in EHV-1 DNA over 21 days was observed at all temperatures conditions assayed. qPCR demonstrated that viral DNA degraded (Fig. 2D), but it is noteworthy that the rate degradation of EHV-1 varied and indicated by a statistically significant interaction between day and temperature (p = 0.006, Supplementary Table 1). EHV-1 DNA degraded more slowly at 4 °C when compared to 20 and 30 °C, over the 21 days of the experiment. In addition, in all samples kept at temperatures ranging from 4–30 °C we could observe CPE in all temperature conditions (Table 2). This result of stability over the 21-day period may reflect the higher input of virus used in this experiment as verified by qPCR results of the stock culture used here (GCN = 14.41). In the previous experiment (serial dilution experiment), we used about 4-fold less virus (GCN = 15.62), this again illustrates that greater viral input increases the time until decay because of the similar decay rate. The results from the qPCR, after cell culture amplification, showed high DNA concentration up to 21 days, suggesting there is robust viral replication (Fig. 3D), however, it also shows after 14 days the DNA degraded more rapidly in higher temperatures (20–30 °C).

### Stability of EHV-1 at different levels of turbidity (sediment concentration)

In the initial experiment, EHV-1 DNA was extracted only from the water phase of the combined water and sediment samples (Fig. 2B). qPCR showed that the EHV-1 DNA concentration varied between the different treatment condition (GCN 3.2 × 10^5^–1.1 × 10^6^) (Fig. 2B); however, DNA was degraded in all sediment conditions over the 21-day experimental period, with sediment having a significant effect on retrievable DNA concentrations (p = 0.006). After the first seven days, the most turbid sample (100 g/L) had higher initial EHV-1 concentrations when compared with the other sediment treatments. However, all turbidity levels showed similar rates of a decline in EHV-1 levels (Fig. 2B). This pattern was also observed in the qPCR after cell culture amplification (Fig. 3B) and plaque assays (Table 2). However, it is important to note that the cell culture samples with higher sediment concentrations (50 g and 100 g), was outcompeted with fungi and bacteria present in the soil. This affected our ability to accurately access the direct impact of the soil at higher concentrations on infectivity of EHV-1. The cell culture results clearly show that the lower amounts of sediment gave rise to plaques up to 14 days, while under higher turbidity conditions, CPE only developed at 1 DPI (Table 2). However, we assume this has more to do with fungal and bacterial growth outcompeting viral replication under cell culture conditions in the laboratory. No statistically significant effect of sediment concentration on EHV-1 DNA stability was observed (p value = 0.363, Supplementary Table 1).

**Figure 4:**
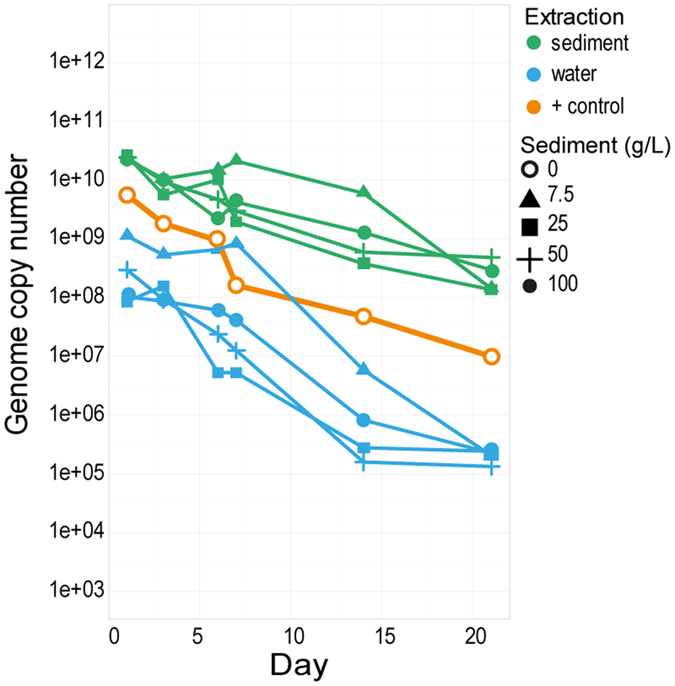
qPCR results for detection of EHV-1 DNA extracted from distilled water (1 L) with sediment (7.5, 25, 50 and 100 g/L) spiked with EHV-1 (10^3.5^ TCID_50_/ml). Samples for DNA extraction were taken from both the sediment (green) and the water (blue). The experiment was run over 21 days at room temperature and samples at six different time point (days). Yellow lines indicate the positive control of EHV-1 in distilled water. Detection limit of EHV-1 is represented with a dotted grey line (~700). All values shown represent GCN of EHV-1.

The second sediment and water experiment (Fig. 4) measured recoverable DNA from sediment and water separately. Surprisingly, EHV-1 concentrations from the extracted sediment were higher than the corresponding water values for all 21 days of the experiment. EHV-1 concentrations in both sediment and water samples were initially high (GCN 8.5 × 10^7^–2.6 × 10^10^). However, after 21days, EHV-1 DNA yields from sediment were significantly higher than from water. The statistical analysis shows that there is a significant difference in the EHV-1 DNA detected in the sediment and water over the 21 days (p < 0.001 in both cases, Supplementary Table 1); with more EHV-1 DNA consistently detected in sediment in all treatments over the 21 days (Fig. 4).

## Discussion

We have demonstrated that EHV-1 can remain infectious in water under different conditions of salinity, different pH, temperature and turbidity conditions over 21 days. Our results suggest that high pH increases the time that EHV-1 remains infectious, while salinity does not demonstrate a significant effect on the persistence or infectivity of EHV-1. Therefore, this could indicate that alkaline water sources may be the likeliest sources for EHV-1 infection. These findings are similar to what has been observed for avian influenza viruses in terrestrial environments, with the viruses favoring water with a slightly basic pH (7.4–8.2) pH, freshwater to brackish salinities (0–20,000 pp)^14^,^22^. Our results also indicate that EHV-1, when shed into aquatic environments, may physically associate with sediment where it may remain stable at a higher concentration than in the water.

The viral dilutions containing the higher virus titers were present longer in water, as the two samples with the highest viral titer (10^−1^ and 10^−2^) produced CPE after fourteen days of incubation in water at room temperature. EHV-1 tenacity was significantly longer in water with higher pH (pH 8–10) (Fig. 1A,B) both in DNA extracted from water and from cell culture. Plaque formation was still visible after treatment at the higher pH conditions after 14 days. This result was similar to what was observed by Doll *et al*.^23^ who demonstrated that EHV-1 remains infectious up to 21 days in pH 8 between 20–27 °C^23^. However, due to the relatively high viral titer necessary for the current experiments, we were unable to draw an accurate link between GCN and plaque forming units (PFUs). The result of EHV-1 being stable and resistant under high pH conditions has been previously shown for other human pathogens, such as norovirus^24^, which is able to survive high pH ranges despite not having an envelope. The enveloped avian influenza was also reported to be more stable in slightly basic conditions (pH 7.4–8.2)^14^. In contrast, herpes simplex virus (HSV) only remains infectious for 4.5 hours under high pH conditions^25^. For equid species these results could suggest transmission of EHV-1 in water would be more likely to occur in alkaline water sources that are accessed by many different individuals, increasing the likelihood that a higher titre of virus particles are maintained in the water. This suggests there are strong differences among herpesviruses in their ability to remain infectious in water with varying pH levels. However, a dilution experiment of a gammaherpesvirus (EHV-2) in distilled and tap water yielded results similar to those observed for EHV-1 (data not shown). This suggests that EHVs may have similar stability in water but that this propensity may not extend to all herpesviruses. From the results, we can speculate that alkaline water sources, often found in places such as Mongolia, China, and Africa^26^,^27^,^28^,^29^, would be best suited to serve as sources of transmission for EHVs. Of note, all three countries have wild equid species (*Equus hemionus hemionus* or Mongolian wild asses in Mongolia and three zebra species, *E. grevi, E. zebra* and *E. quagga* in Africa). In all three countries, equids and sympatric species are subject to seasonal water shortages and congregate at high density in mixed species assemblages at residual water holes.

While there is a trend showing that higher salinity resulted in EHV-1 DNA remaining stable after seven days while lower salinity levels decreased stability over 21 days (Figs 2A and 3A) (Table 1) when compared with the positive controls; there were no statistically significant effects of salinity for either qPCR post cell culture or the cell culture results. Therefore, despite the effect on DNA stability, we could not detect a statistically significant effect on infectivity. Although the effect of salinity on herpesviruses has not been previously investigated, studies of avian influenza virus under different salinity conditions in water have demonstrated that they are preferentially stable in fresh to brackish water environments (0–20,000 ppm)^14^. While DNA stability of EHV-1 was highest between 3,000 to 35,000 ppm after seven days, all concentrations negatively impacted the infectivity of EHV-1 Other studies have demonstrated that phosphate-buffered saline solutions enabled the survival of pseudorabies virus for up to ten days, compared to two days in lagoon water^30^. The data overall suggest that a wide range of salt concentrations will have limited impact on EHV infection but that high salinity preserves viral DNA at the expense of viral infectivity. However, the water sources used by both captive and wild equids are unlikely to have salinity levels equivalent to those that inhibit infectivity and thus EHV is likely environmentally stable across a broad range of salt conditions in nature.

Temperature had a significant effect on the persistence of EHV-1 in water (Fig. 2, Supplementary Table 1) suggesting that EHV-1 may remain infectious for longer at colder water temperatures (4 °C) This result is consistent with what was previously reported by Doll *et al*.^23^ which found that inactivation of the virus was more rapid between 20–27 °C than if stored at 4 °C^23^. Remarkably, EHV-1 could be isolated in cell culture for up to 21 days under all treatment conditions, indicating that EHV-1 is able to remain infectious for extended periods of time and in a wide temperature range of 4–30 °C (Table 2). This result, however, differed from the original results from the serial dilution experiment, in which only the sample with the highest viral load induced CPE up to 14 days of treatment. We suspected that the difference in outcome may have been a result of inaccurate TCID_50_ calculations, which was further confirmed through qPCR results showing an about 4-fold difference between two different viral stocks in these experiments. This result could hint a threshold phenomenon in the sense that a certain amount of infectious virus is necessary for sustained infectivity over longer time scales. Notably, the stability of EHV-1 over an ambient temperature range (4–20 °C) is similar to the water-borne viral haemorrhagic septicaemia virus (VHSV) that remained stable up to 13 days in 15 °C in freshwater^31^. However, EHV-1 is able to remain infectious for over two weeks in water of higher temperatures, whereas VHSV can only remain infectious for two days at 30 °C.

From the results, we surmise that EHV-1 may remain stable and infectious in a range of water sources where temperatures may vary considerably. The stability of the virus across a range of water temperature increases the opportunity for EHV-1 to be transmitted in many different environments and may suggest transmission is possible across different seasons.

Our results suggest that turbidity may influence the stability of EHV-1. The amount of sediment in the water had a statistically significant effect on the rate of DNA degradation (Figs 2B and 3B Supplementary Table 1). It was surprising to us that EHV-1 DNA degraded more rapidly in the water than in the sediment. This suggests that the soil may interact with the EHV-1 particles, drawing the virus from the water to the sediment, where it may be protected. It may be possible that viral envelope glycoproteins interact with charged molecules in the soil and protect the virus hydrolytic degradation. Sediment may also protect viruses from UV light, particularly in shallow water, which is known to cause significant degradation of many viruses^32^,^33^. However, this warrants further investigation.

Water sources in many parts of the world are frequently accessed by different equid species and non-equids living in sympatry^1^. In Mongolia and Africa, many lentic water sources are often non-perennial and frequented by different species of animals that congregate together, which is more prevalent in times of seasonal water shortages. During the dry seasons, both large mammalian predators and prey are often observed drinking together in close proximity^34^,^35^,^36^. It is at these water sources that equid species infected with EHV-1 could potentially be shedding virus into the water where it can persist long after the animals have moved on. As this study has demonstrated EHV-1 to be stable under different environmental water conditions, the water sources would provide EHV-1 with the opportunity to infect potential hosts sharing the water source. This in turn may account for the recent reports of EHV-1-like viruses detected in non-equid species in captivity, as water is a potential transmission conduit for the virus between different species^4^,^5^,^6^,^7^,^8^,^9^.

Taken together, this is the first study using both traditional and molecular techniques examining the persistence and infectivity of EHV-1in water under different conditions that likely vary in the environment where equids are found. EHV-1 was shown to be stable and infectious for over a week in all experiments conducted and up to three weeks under some conditions suggesting that EHV-1 remains infectious in water. Although our experiments do not mimic EHV-1 in the natural environment, they provide valuable insights into factors that may influence their stability and ability to remain infectious in water. Further research is necessary to establish whether the laboratory results reflect virus biology in environmental samples.

## Additional Information

**How to cite this article**: Dayaram, A. *et al*. Long term stability and infectivity of herpesviruses in water. *Sci. Rep.* **7**, 46559; doi: 10.1038/srep46559 (2017).

**Publisher's note:** Springer Nature remains neutral with regard to jurisdictional claims in published maps and institutional affiliations.

## Supplementary Material

Supplementary Information

## Figures and Tables

**Table 1:** Cytopathic effects (CPE) observed in cell culture after five days.

	10^−1^	10^−2^	10^−3^	10^−4^	10^−5^	10^−6^	10^−7^	10^−8^	10^−9^
Day 1	+	+	+	−	−	−	−	−	−
Day 3	+	+	+	−	−	−	−	−	−
Day 6	+	+	−	−	−	−	−	−	−
Day 7	+	+	−	−	−	−	−	−	−
Day 14	+	+	−	−	−	−	−	−	−
Day 21	−	−	−	−	−	−	−	−	−

Distilled H_2_O (1 L) spiked with a 10-fold serial dilution of EHV-1 (10^3.5^ TCID_50_/ml). Run over 21 days are sampled at six different points (days). ^+^denotes positive for any CPE in cell culture. ^−^denotes negative for any CPE in cell culture.

**Table 2:** Cytopathic effects (CPE) observed in cell culture after five days.

	Day 1	Day 3	Day 6	Day 7	Day 14	Day 21
Sediment 7.5 g	+	+	+	+	+	−
Sediment 25 g	+	+	+	+	+	−
Sediment 50 g	+	−	−	−	−	−
Sediment 100 g	+	−	−	−	−	−
pH 4	−	−	−	−	−	−
pH 6	+	+	−	−	−	−
pH 8	+	+	+	+	+	−
pH 10	+	+	+	+	+	+
Salinity 0.05 g	+	−	−	−	−	−
Salinity 1.0 g	+	+	+	+	+	−
Salinity 3.0 g	+	+	+	+	+	+
Salinity 35.0 g	+	+	+	+	+	−
Temperature 4 °C	+	+	+	+	+	+
Temperature 20 °C	+	+	+	+	+	+
Temperature 30 °C	+	+	+	+	+	+

Samples include distilled H_2_O (1 L) with treatments sediment (7.5, 25, 50, and 100 g/L), pH (4,6,8,10), salinity (0.05, 1.0, 3.0, and 35 g/L) and temperature (4, 20 and 30 °C) spiked with EHV-1 (10^3.5^ TCID_50_/ml). Run over 21 days are sampled at six different points (days). ^+^denotes positive for any CPE in cell culture. ^−^denotes negative for any CPE in cell culture.
