# Supplementary material for: Long term stability and infectivity of herpesviruses in water
Source: Sci Rep. 2017 Apr 21;7:46559. doi: 10.1038/srep46559 (PMC5399353; doi:10.1038/srep46559)
Supplement: Supplementary Information [file srep46559-s1.pdf]

Long term stability and infectivity of herpesviruses in water

Anisha Dayaram[a*], Mathias Franz[a], Alexander Schattschneider[a], Armando M. Damiani[b], Sebastian Bischofberger[b], Nikolaus Osterrieder[b], Alex D. Greenwood[a,c]

[a] Leibniz-Institute for Zoo and Wildlife Research, Alfred-Kowalke-Strasse 17, 10315 Berlin, Germany
[b] Institut für Virologie, Freie Universität Berlin, Robert-von-Ostertag-Str. 7-13, 14163 Berlin, Germany
[c] Department of Veterinary Medicine, Freie Universität Berlin, Oertzenweg 19b, 14163, Germany

Supplementary Table 1: Results of statistical analyses performed on copy number values and cytopathic effects (CPE) observed in cell culture. The response variables were: dilution factor, day, pH, salinity, temperature, and sediment. The predictors of copy number were log transformed: Initial DNA extraction A, B, C D, F, G. Cell culture copy number H, I, J, K and M. Cell culture values (no transformation) E, L, M and N.

A EHV-1 serial dilution: copy number initial water

	Estimate	Std. Error	t-value	p-value	Pr(>t)
Intercept	20.96	0.75	27.76	0.00	***
Log (Dilution factor)	-0.62	0.05	-11.73	0.00	***
Day	-0.42	0.07	-6.20	0.00	***
Log (Dilution factor):Day	0.01	0.00	3.04	0.00	**

B EHV-1 salinity: copy number initial water

	Estimate	Std. Error	Error	p-value	Pr(>t)
Intercept	22.37	0.41	54.59	0.00	***
Salinity	0.02	0.02	0.96	0.35	
Day	-0.38	0.03	-10.96	0.00	***

C EHV-1 sediment: copy number initial water

	Estimate	Std. Error	t-value	p-value	Pr(>t)
Intercept	21.98	0.51	43.49	0.00	***
Soil	-0.03	0.01	-4.74	0.00	***
Day	-0.40	0.04	-10.29	0.00	***

D EHV-1 pH: copy number initial water

	Estimate	Std. Error	t-value	p-value	Pr(>t)
Intercept	18.02	0.96	18.79	0.00	***
pH	0.44	0.12	3.55	0.00	**
Day	-0.35	0.04	-9.66	0.00	***

E EHV-1 Salinity: cell culture (CPE)

	Estimate	Std. error	z value	p value	Pr(>t)
Intercept	2.02	0.94	2.14	0.03	*
Salinity	0.05	0.04	1.05	0.29	
Day	-0.15	0.08	-1.98	0.05	*

F EHV-1 temperature: copy no. initial water (response log transformed)

	Estimate	Std. Error	z value	p-value	Pr(>t)
Intercept	3.25	0.03	118.47	0.00	***
Temperature	0.00	0.00	-0.10	0.92	
Day	-0.01	0.00	-3.34	0.01	**
Temperature:Day	0.00	0.00	-3.21	0.01	**

G EHV-1 temperature: copy no. cell culture (response log transformed)

	Estimate	Std. Error	z value	p-value	Pr(>t)
Intercept	3.40	0.03	135.85	0.00	***
Temperature	0.00	0.00	-0.38	0.71	
Day	0.00	0.00	-1.50	0.16	

*Indicates the p and z value level of significance

H EHV-1 serial dilution: copy number cell culture

	Estimate	Std. Error	t-value	p-value	Pr(>t)
Intercept	3.56	0.08	47.07	0.00	***
Log (Dilution factor)	-0.05	0.01	-9.75	0.00	***
Day	-0.05	0.01	-7.37	0.00	***
Log (Dilution factor):Day	0.00	0.00	2.66	0.01	*

I EHV-1 salinity: copy number cell culture

	Estimate	Std. Error	Error	p-value	Pr(>t)
Intercept	3.04	0.13	22.67	0.00	***
Salinity	0.00	0.01	0.42	0.68	
Day	-0.04	0.01	-3.84	0.00	***

J EHV-1 sediment: copy number cell culture

	Estimate	Std. Error	t-value	p-value	Pr(>t)
Intercept	2.80	0.18	15.74	0.00	***
Soil	-0.01	0.00	-3.42	0.00	**
Day	-0.04	0.01	-2.59	0.02	*

K EHV-1 pH: copy number cell culture

	Estimate	Std. Error	t-value	p-value	Pr(>t)
Intercept	1.99	0.44	4.52	0.00	***
pH	0.13	0.06	2.24	0.03	*
Day	-0.05	0.02	-3.16	0.00	**

L EHV-1 sediment: cell culture (CPE)

	Estimate	Std. Error	z value	p value	Pr(>t)
Intercept	-3.24	2.40	-1.35	0.18	
sediment	-0.04	0.02	-1.91	0.06	
Day	0.30	0.16	1.91	0.06	

M EHV-1 pH: cell culture (CPE)

	Estimate	Std. Error	z value	p-value	Pr(>t)
Intercept	-9.51	3.77	-2.52	0.01	*
pH	1.43	0.56	2.55	0.01	*

N EHV-1 copy number sediment vs water

	Estimate	Std. Error	t-value	p-value	Pr(>t)
Intercept	23.13	0.49	46.99	0.00	***
Concentration	0.01	0.01	1.43	0.16	
Extractionwater	-1.88	0.70	-2.70	0.01	**
Day	-0.24	0.04	-6.25	0.00	***
Concentration:Extractionwater	-0.04	0.01	-3.55	0.00	***
Extractionwater:Day	-0.14	0.05	-2.56	0.01	*

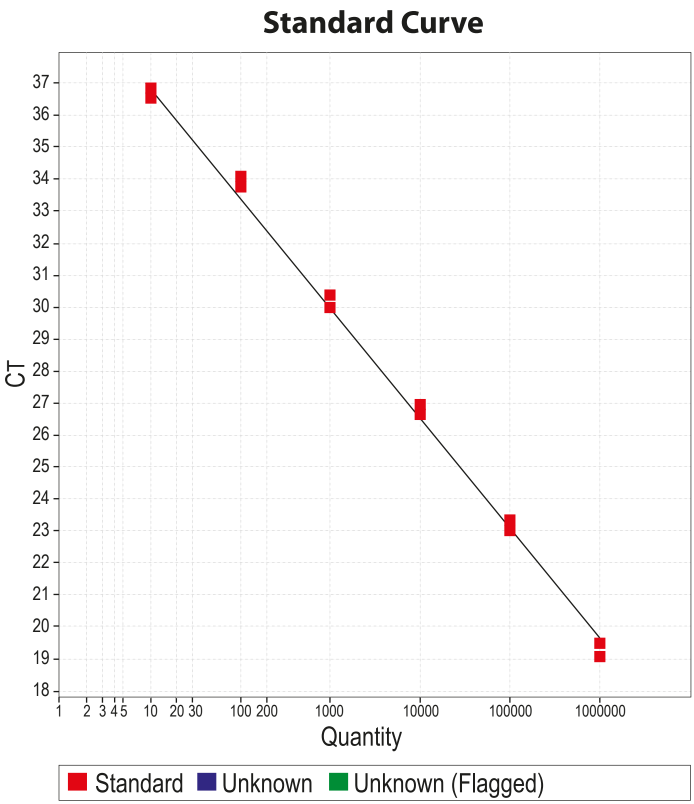

Supplementary Figure 1: qPCR standard curve generated from a six fold serial dilution of plasmid DNA from EHV-1 gB glycoprotein BAC, Ab4 strain.
